# Increased e-cigarette use prevalence is associated with decreased smoking prevalence among US adults

**DOI:** 10.1186/s12954-024-01056-0

**Published:** 2024-07-18

**Authors:** Floe Foxon, Arielle Selya, Joe Gitchell, Saul Shiffman

**Affiliations:** Pinney Associates, Inc, 201 North Craig Street, Suite 320, Pittsburgh, PA 15213 USA

**Keywords:** Adult, E-cigarette, Vaping, Counterfactual, NHIS, Smoking, Trend modelling

## Abstract

**Background:**

If US adults who smoke cigarettes are switching to e-cigarettes, the effect may be observable at the population level: smoking prevalence should decline as e-cigarette prevalence increases, especially in sub-populations with highest e-cigarette use. This study aimed to assess such effects in recent nationally-representative data.

**Methods:**

We updated a prior analysis with the latest available National Health Interview Survey data through 2022. Data were cross-sectional estimates of the yearly prevalence of smoking and e-cigarette use, respectively, among US adults and among specific age, race/ethnicity, and sex subpopulations. Non-linear models were fitted to observed smoking prevalence in the pre-e-cigarette era, with a range of ‘cut-off’ years explored (i.e., between when e-cigarettes were first introduced to when they became widely available). These trends were projected forward to predict what smoking prevalence would have been if pre-e-cigarette era trends had continued uninterrupted. The difference between actual and predicted smoking prevalence (‘discrepancy’) was compared to e-cigarette use prevalence in each year in the e-cigarette era to investigate whether the observed decline in smoking was statistically associated with e-cigarette use.

**Results:**

Observed smoking prevalence in the e-cigarette era was significantly lower than expected based on pre-e-cigarette era trends; these discrepancies in smoking prevalence grew as e-cigarette use prevalence increased, and were larger in subpopulations with higher e-cigarette use, especially younger adults aged 18–34. Results were robust to sensitivity tests varying the analysis design.

**Conclusions:**

Population-level data continue to suggest that smoking prevalence has declined at an accelerated rate in the last decade in ways correlated with increased uptake of e-cigarette use.

**Supplementary Information:**

The online version contains supplementary material available at 10.1186/s12954-024-01056-0.

## Background

The introduction of e-cigarettes in the US in 2007 and their significant uptake among adults since 2010 [1] may have impacted the prevalence of combustible cigarette smoking in the following ways:

(1) If e-cigarettes act as a ‘gateway’ to cigarette smoking, then at the population level, increased e-cigarette use prevalence would coincide with increased smoking prevalence [2, 3], or at the very least, a slowing of the rate of decline of smoking prevalence. While it is often observed that youth who use e-cigarettes are more likely to later initiate cigarette smoking, research has questioned whether this effect is causal, rather than due to shared risk factors or common liabilities between e-cigarette use and smoking [4–8]. Evidence for the ‘gateway’ effect has not been detected in population-level studies on prevalence of e-cigarette use and smoking among young people [9–18]; indeed, smoking prevalence remains at an all-time low among US adolescents [19, 20] and young adults [21], despite increases in e-cigarette use.

(2) If e-cigarettes help adults who smoke combustible cigarettes to switch away from cigarette smoking (i.e., ‘switching’), and/or if they divert individuals who otherwise would have started smoking away from taking up cigarettes in the first place (i.e., ‘diversion’), then at the population level, increased e-cigarette use prevalence would coincide with greater decreases in smoking prevalence than would otherwise be expected.

(3) Because effects (1) and (2) are not mutually exclusive, both could occur simultaneously, their balance leading to a ‘net’ gateway, switching/diversion, or mutual cancellation.

The net impact of the introduction and increased use of e-cigarettes on smoking at the US-population-level is debated. The present study is an update on a previous analysis modeling population-level prevalence [22] that assessed whether and how much the introduction of e-cigarettes in the US may be correlated with declining smoking prevalence among populations of US adults using the National Health Interview Survey (NHIS). In examining this association, analyses consider particular subpopulations by age, sex, and race/ethnicity. These are relevant both to establish the robustness of observed effects, and because the relation between e-cigarette use and smoking would be expected to be strongest in subpopulations with higher e-cigarette use prevalence, and, indeed, absent in strata with very low e-cigarette use. These particular subpopulations are relevant because previous NHIS data have shown substantial variations in e-cigarette use prevalence between them. For example, Cornelius et al. [21] reported that current e-cigarette use prevalence in 2021 was more than ten times higher among young adults compared to older adults, and more than two times higher among non-Hispanic White adults compared to non-Hispanic Black adults. Thus, we expect different impacts of e-cigarette use on smoking prevalence at the population level in these demographic subpopulations.

Here, we present additional analyses using the latest-available annual NHIS data (from 1990 to 2022) to update our prior report [22], and address a critique based on the selection of a starting year for the period when e-cigarettes could affect smoking prevalence [23].

## Methods

### Data

Estimates for cigarette smoking prevalence among US adults from 1990 to 2022 were derived from CDC’s annual, cross-sectional, nationally-representative National Health Interview Survey (NHIS [24]; 32 waves; *n* = 17,317–43,732 per wave). Because NHIS underwent a redesign in 2019 [25], and because data collection procedures were impacted by the COVID-19 pandemic from 2020 to 2022 [26–28], a sensitivity test was run in which the 2019–2022 point estimates were removed from the analysis to account for possible variation in results.

Current smoking was defined as having smoked at least 100 cigarettes (lifetime) and ‘now’ smoking cigarettes ‘every day’ or ‘some days’ [29]. NHIS collected data on e-cigarette use prevalence from 2014 to 2022, defining current e-cigarette use as ‘now’ using e-cigarettes ‘every day’ or ‘some days’ [21, 29, 30]. (Note that cumulative lifetime e-cigarette use was not measured in NHIS, so ‘established’ use cannot be assessed). The definition of e-cigarette use in NHIS refers to ‘nicotine’ e-cigarettes and excludes marijuana vaping. Changes in questionnaire wording over time are described in the online supplemental materials.

Current smoking and current e-cigarette use prevalence were estimated across time in three age subpopulations (18–34 years, 35–54 years, and 55 + years, as in Axelsson et al. [31]), three race/ethnicity subpopulations (Hispanic, non-Hispanic (NH) White, and NH Black), and two sex subpopulations (female and male). The selection of these subpopulations maximized sample size in prevalence estimates while still allowing for variation by demographics; non-Hispanic Other race/ethnicity subpopulations could not be analyzed in NHIS due to sample size constraints (some estimates had relative standard errors > 30% which were suppressed due to statistical unreliability, per standard practice [29]).

### Analysis

The transition from the pre-e-cigarette era to the e-cigarette era (i.e., the time before e-cigarettes were introduced or became widely used vs. the time after) was defined by a ‘cut-off’ year that marked the onset of a time when e-cigarette use could have materially affected smoking prevalence. E-cigarettes were first introduced in the US in 2007; current e-cigarette use prevalence remained negligible (rounding to 0%) as late as 2010 [32]; Zhu et al. [1] reported that “use of electronic cigarettes in the USA… became noticeable around 2010”; 2010 was objectively identified as the inflection point or knee of the NHIS data using the ‘Kneedle’ algorithm [22, 33]; financial analyses by Wells Fargo and Agora Financial suggest minimal e-cigarette market presence in the years prior to 2010 [34]; and 2010/2011 has been used as the cut-off year in multiple other cigarette/e-cigarette prevalence modelling analyses [10, 12, 14, 15, 35]. Thus, the current analysis examines all plausible cutoff-years between the pre-e-cigarette and e-cigarette eras (from 2006 to 2011).

Non-linear (exponential decay) weighted least squares models regressed smoking prevalence on year from 1990 to the cut-off year to model smoking prevalence in the pre-e-cigarette era. The resulting model was projected forward in time to predict what smoking prevalence might have been in each year after the cut-off in the absence of e-cigarette use (i.e., if pre-e-cigarette era smoking trends had continued uninterrupted). That analysis was repeated for each cut-off year from 2006 to 2011 in separate models.

For each year after the cut-off, the actual NHIS-measured smoking prevalence was subtracted from the predicted (i.e., projected or modelled) smoking prevalence to define the ‘discrepancy’ in cigarette smoking prevalence (i.e., the difference between what smoking prevalence might have been had prior trends continued, and the actual survey-measured smoking prevalence in the presence of e-cigarette use). Positive discrepancy values indicate that actual smoking prevalence was lower than expected.

Pearson correlation coefficients between current e-cigarette use prevalence and smoking discrepancy were estimated, and two-tailed p-values computed (alpha = 0.05). Because NHIS only began measuring prevalence of e-cigarette use in 2014, following Pesola et al. [36] we imputed e-cigarette use prevalence in years prior to 2014 using available data (2014-on) by assuming linear growth in e-cigarette use from the cut-off year through 2014. A sensitivity analysis tested the impact of this imputation by excluding imputed e-cigarette use.

These analyses were repeated for each of the age (18–34, 35–54, 55+), race/ethnicity (Hispanic, NH White, NH Black), and sex (female, male) subpopulations to investigate demographic variation in associations between cigarette smoking and e-cigarette use. We conservatively considered an association between smoking and e-cigarette use prevalence in a given subpopulation to be significant only if the association was statistically significant across all sensitivity tests.

We used procedures for complex surveys to estimate standard errors for the NHIS prevalence estimates (as in CDC analyses of NHIS data [21, 29, 30]). Weighted models were used to give the greatest weight to estimates with the least uncertainty. Root Mean Square Errors (RMSE) were calculated to assess goodness of fit, as appropriate for forecasts on means [37].

NHIS point estimates were calculated in SAS version 9.4. All other analyses were performed in Python version 3.11.5 with the packages NumPy version 1.24.3, Scipy version 1.11.1, Uncertainties version 3.1.7, and Matplotlib version 3.7.2.

## Results

The NHIS sample distribution across all years by demographic and tobacco product use status is shown in Table 1. The sample (*N* = 959,353 observations) is roughly evenly distributed by age subpopulation. Reflecting the US population, the sample is majority NH White and majority never smoking/e-cigarette using.

**Table 1:** Combined sample characteristics

Demographic		Weighted Percent of sample % (*n*)
Total		*N* = 959,353
Age	18–34	31.5 (270,383)
	35–54	36.4 (335,689)
	55+	32.1 (353,281)
Race/Ethnicity	Hispanic	12.8 (136,511)
	NH White	70.1 (618,667)
	NH Black	11.5 (125,571)
	NH Other	5.6 (49,896)
Sex	Female	51.9 (537,340)
	Male	48.1 (422,003)
Cigarette Smoking Status	Current	19.7 (190,678)
	Former	22.5 (218,464)
	Never	57.9 (540,216)
E-Cigarette Use Status	Current	3.9 (9,266)
	Former	12.0 (30,383)
	Never	84.1 (231,334)

**Fig. 1:**
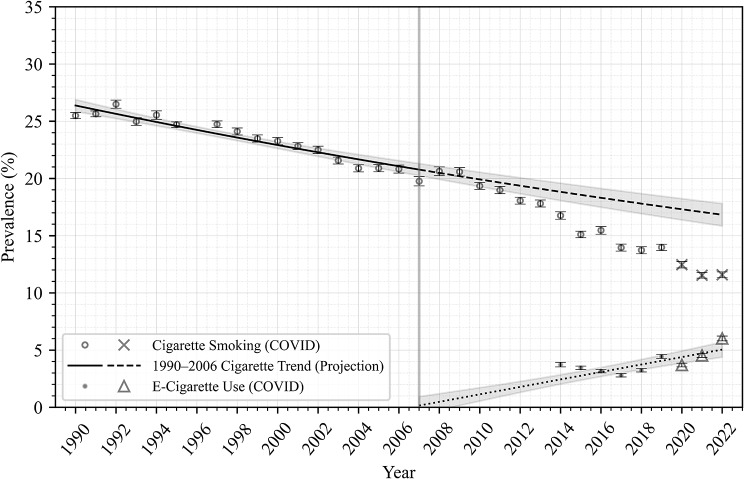
Cigarette smoking and E-Cigarette use prevalence (All Adults)

**Fig. 2:**
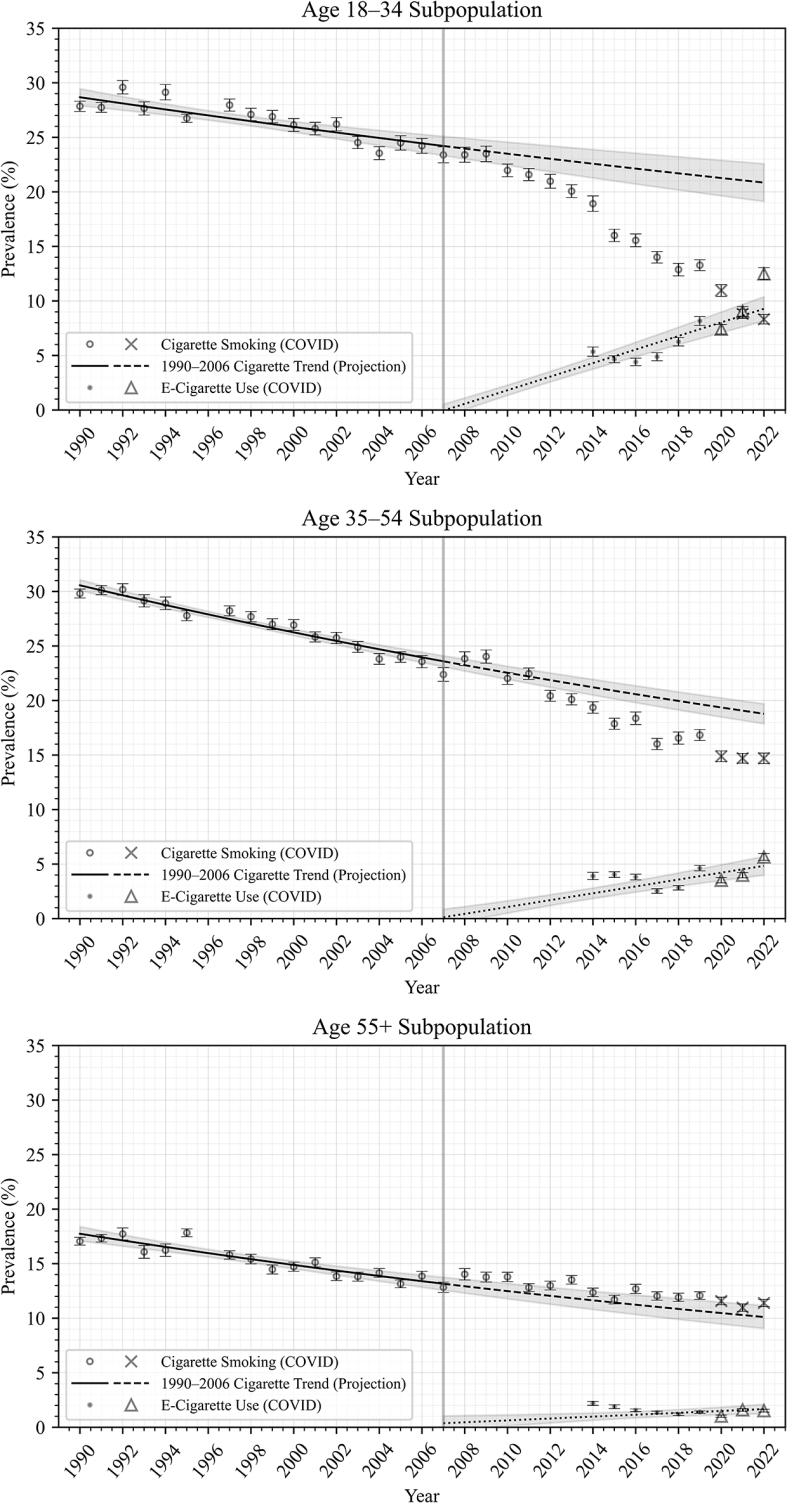
Cigarette smoking and E-Cigarette use prevalence by age

**Fig. 3:**
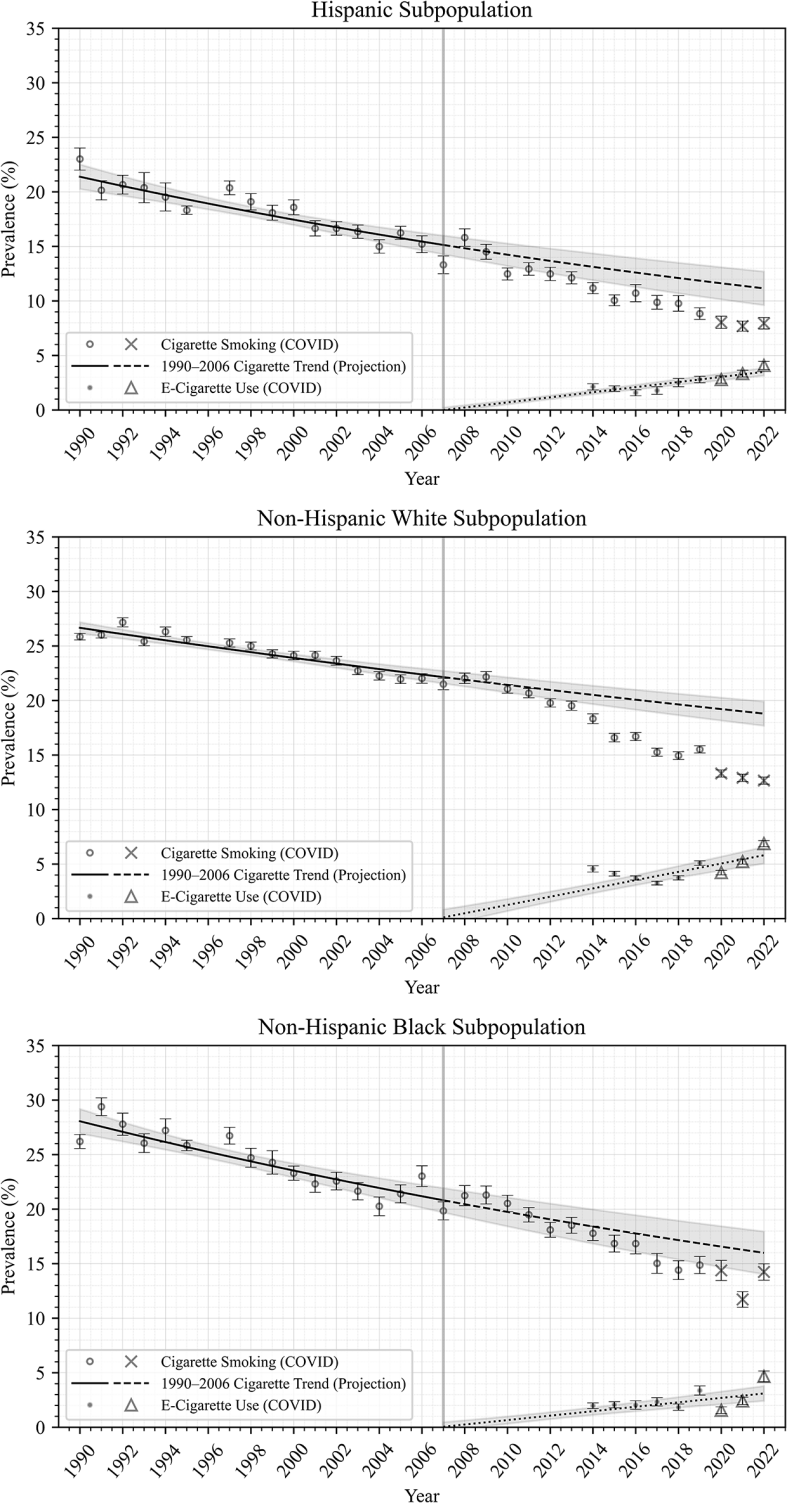
Cigarette smoking and E-Cigarette use prevalence by race/ethnicity

**Fig. 4:**
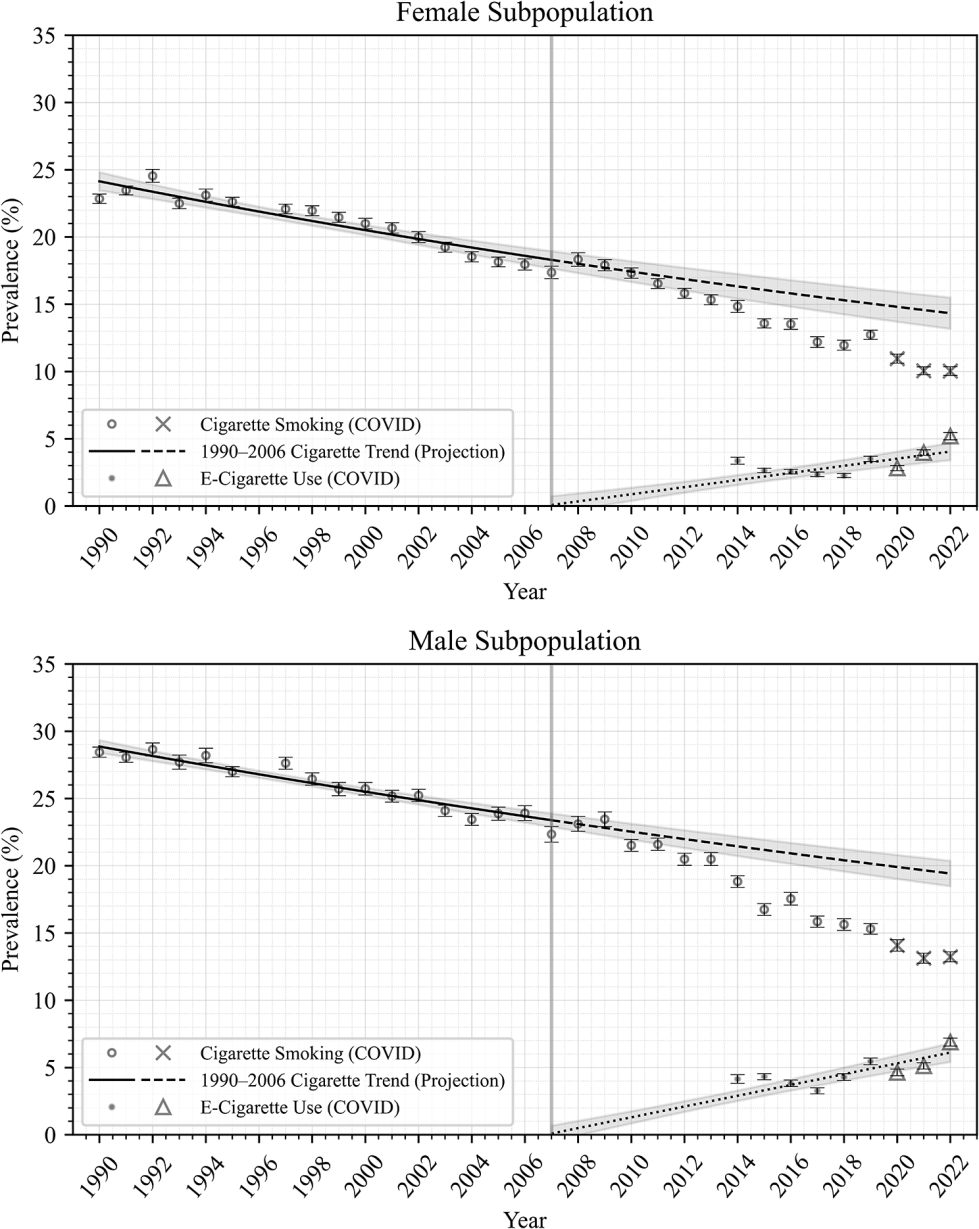
Cigarette Smoking and E-Cigarette Use Prevalence by Sex

Figures 1, 2, 3 and 4 show trends in prevalence of cigarette smoking and e-cigarette use in the NHIS data, as well as modeled data, among all adults (Fig. 1) and in subpopulations by age (Fig. 2), race/ethnicity (Fig. 3), and sex (Fig. 4). Each point estimate from NHIS data is plotted with error bars, and 95% confidence intervals are plotted around the projected trends, displaying the uncertainties in the modeled estimates. In general, these figures show that smoking prevalence declined steadily from 1990 to 2006 among all adults and among each subpopulation analyzed. Model RMSEs ranged from 0.5 to 1.1, which are low relative to the y-axis range, suggesting good fit to historical NHIS data across the period 1990–2006, as evident in the figures.

After the period 1990–2006, smoking prevalence apparently underwent an accelerated decline among all adults (Fig. 1) and among the younger adults (age 18–34; Fig. 2), Hispanic and non-Hispanic White adults (Fig. 3), and both male and female subpopulations (Fig. 4), such that observed smoking prevalence in these populations was *lower* than expected based on 1990–2006 trends (dashed lines).

E-cigarette use prevalence was highest among the younger adult (18–34) subpopulation, and lowest among the older adult (55+) subpopulation (Fig. 2); was higher among non-Hispanic White adults compared to Hispanic adults and non-Hispanic Black adults (Fig. 3); and was slightly higher among males compared to females (Fig. 4).

As seen in Table 2, a statistically significant association between smoking prevalence discrepancy (i.e., the degree to which actual prevalence fell below the expected prevalence) and e-cigarette use prevalence across all sensitivity tests was identified for the total adult population, as well as in the younger adult (age 18–34), Hispanic, non-Hispanic White, male, and female subpopulations. Associations were not statistically significant across all sensitivity tests for the non-Hispanic Black and older adult subpopulations. Associations were strongest in subpopulations with the greatest e-cigarette use prevalence, e.g. Pearson correlation coefficients were high (consistently ranging from 0.8 to 0.9) and statistically significant (ps ≤ 0.01) across sensitivity tests for the younger adult subpopulation; but were low (-0.4–0.4) and non-significant (ps > 0.05) for the older adults age 55 + subpopulation, consistent with expectations.

**Table 2:** Correlation between Smoking Discrepancy and E-Cigarette Use Prevalence

Subpopulation		Sensitivity Test with Alternative Cut-Off Year Pearson r* (*p* value)						Sensitivity Test Excluding 2019–2022 Point Estimates (Using 2007 as Cut-Off Year†) Pearson r* (*p* value)	Sensitivity Test Excluding Imputed E-Cigarette Prevalence (Using 2007 as Cut-Off Year†) Pearson r* (*p* value)
		2006	2007†	2008	2009	2010	2011		
All Adults (Total)		**0.9 (< 0.001)**	**0.9 (< 0.001)**	**0.9 (< 0.001)**	**0.9 (< 0.001)**	**0.9 (< 0.001)**	**0.8 (< 0.001)**	**0.8 (0.001)**	**0.8 (0.01)**
Age	18–34	**0.9 (< 0.001)**	**0.9 (< 0.001)**	**0.9 (< 0.001)**	**0.9 (< 0.001)**	**0.9 (< 0.001)**	**0.9 (< 0.001)**	**0.9 (< 0.001)**	**0.9 (< 0.001)**
	35–54	**0.8 (< 0.001)**	**0.8 (< 0.001)**	**0.8 (< 0.001)**	**0.8 (0.001)**	**0.7 (0.005)**	**0.7 (0.02)**	**0.7 (0.01)**	0.4 (0.2)
	55+	-0.2 (0.5)	-0.1 (0.8)	0.3 (0.3)	0.3 (0.3)	0.4 (0.2)	0.3 (0.3)	0.0 (1.0)	-0.4 (0.2)
Race/Ethnicity	Hispanic	**0.8 (< 0.001)**	**0.8 (< 0.001)**	**0.9 (< 0.001)**	**0.9 (< 0.001)**	**0.9 (< 0.001)**	**0.9 (< 0.001)**	**0.7 (0.02)**	**0.8 (0.01)**
	NH White	**0.9 (< 0.001)**	**0.9 (< 0.001)**	**0.9 (< 0.001)**	**0.9 (< 0.001)**	**0.9 (< 0.001)**	**0.8 (< 0.001)**	**0.8 (< 0.001)**	**0.7 (0.01)**
	NH Black	**0.6 (0.008)**	**0.6 (0.02)**	**0.6 (0.01)**	**0.6 (0.02)**	0.6 (0.05)	0.5 (0.1)	**0.7 (0.01)**	0.2 (0.6)
Sex/Gender	Female	**0.8 (< 0.001)**	**0.8 (< 0.001)**	**0.9 (< 0.001)**	**0.8 (< 0.001)**	**0.8 (< 0.001)**	**0.8 (0.004)**	**0.7 (0.005)**	**0.7 (0.04)**
	Male	**0.9 (< 0.001)**	**0.9 (< 0.001)**	**0.9 (< 0.001)**	**0.9 (< 0.001)**	**0.9 (< 0.001)**	**0.9 (< 0.001)**	**0.9 (< 0.001)**	**0.8 (0.004)**

*Pearson correlation coefficient between smoking discrepancy and e-cigarette use prevalence

† Analysis shown in the figures

Bold indicates statistical significance (*p* < 0.05)

## Discussion

This research examined whether observed trends in smoking prevalence among US adults during the e-cigarette era were consistent with empirically-derived projections based on trends before the e-cigarette era, and whether any discrepancy between observed and expected smoking prevalence was correlated with e-cigarette use prevalence, and thus might be explained by e-cigarette use. Significant discrepancies in smoking prevalence were identified, such that observed smoking prevalence in the e-cigarette era was lower than was to be expected based on pre-e-cigarette era trends, i.e., actual smoking prevalence was lower than it otherwise would have been if trends from before e-cigarettes were introduced or became prevalent had continued uninterrupted. These discrepancies were greatest for subpopulations with greatest e-cigarette use prevalence, especially younger adults (18–34). Findings were supported by sensitivity tests and were particularly robust to the choice of cutoff year marking the beginning of the e-cigarette era, giving confidence to the results reported.

Some of the observed smoking discrepancy is likely attributable to other major national smoking interventions, namely the FSPTCA and CDC’s ‘Tips^®^’ campaign. However, even very optimistic estimates of the FSPTCA and Tips^®^ campaign effects *combined*, based on published estimates of the effects of these interventions [38, 39], are unable to fully explain the observed smoking prevalence in NHIS (see online supplemental materials). Thus, criticisms arguing that other factors alone besides e-cigarette use sufficiently account for observed declines in smoking prevalence [40] are contradicted by these analyses, leaving room for a potential effect by the observed association between increasing e-cigarette use and decreasing smoking.

The previous version of this article was criticized with respect to the decision to impute e-cigarette use linearly from the cut-off year to 2014 [40], a technique also used by Pesola et al. [36]. Imputation is justified in these analyses on two grounds: first, it is clear from the figures provided that linear imputation fits the e-cigarette use data reasonably well (i.e., overlapping confidence estimates for survey-measured e-cigarette use prevalence and the linear interpolation line), and second because observed associations between decreased smoking prevalence and increased e-cigarette use remain consistent in sensitivity tests excluding the linearly-imputed e-cigarette prevalence. Furthermore, it is not clear as to what if any alternative method besides linear interpolation would be appropriate for imputing these data. Thus, criticism of the imputation procedure based on arguments that these linear interpolations do not fit the data or that such imputations alter the findings [40] are also contradicted by these analyses.

Similarly, concerns were raised about the choice of ‘cut-off’ year that marked the onset of a time when e-cigarette use could have materially affected smoking prevalence [40]. The present study explored a range of cut-off years from 2006 to 2011 and results were not sensitive to the choice of cut-off year across this range, suggesting oppositely to the critique that the findings are robust.

The observed association between increasing e-cigarette use prevalence and decreasing smoking prevalence suggests a possible population-level displacement of combustible cigarettes by e-cigarettes. This finding is consistent with many other population-level studies across a range of modelling techniques, including agent-based models [15], dynamical system models [14, 35, 41–43], time-series regression models [36, 44], and other techniques [1, 45]. Those studies have similarly concluded that increased e-cigarette use is associated with more rapid declines in smoking prevalence than would otherwise be expected.

The findings of the present study are also consistent with longitudinal cohort studies and naturalistic clinical trials, in which high rates of switching away from smoking, reduced cigarette consumption, and minimal smoking initiation/relapse have been observed in individuals using particular e-cigarette products in real-world settings [46–50]; as well as econometric research on product substitution between cigarettes and e-cigarettes and their cross-price elasticities [51–57]. Randomized controlled trials have also shown that use of e-cigarettes can result in discontinuation of smoking [58–60]. According to other CDC survey data, switching completely to e-cigarettes is now a more popular stop-smoking method among US adults than nicotine patches/gum, FDA-approved medications such as varenicline and bupropion, getting help from a doctor or other health professional, telephone quit lines, and other methods [43, 72]﻿.

These findings are not consistent with a net ‘gateway’ effect from prior e-cigarette use to subsequent smoking; if a net gateway were present, then observed smoking prevalence in the e-cigarette era would be the same as or greater than expected based on pre-e-cigarette era trends. That is, the correlations would be null or negative, rather than the strongly and significantly positive correlations we observed.

It is not surprising that we did not observe an association among older adults in the present study, since older adults have generally not adopted e-cigarettes (e.g., see Figs. 2 and [21, 61]), precluding any effect on their smoking. This finding is also consistent with longitudinal cohort studies which show that age is significantly associated with switching [46].

Although population-level evidence continues to suggest a potential displacement of smoking by e-cigarette use, possibly even in diverting individuals from initiation, use of e-cigarettes by unintended subpopulations (e.g., nonsmokers) remains a high concern; efforts to reduce e-cigarette use by unintended subpopulation must continue to be prioritized while simultaneously allowing adults who smoke to access reduced-risk products.

It is informative to compare the effects observed among adults in the present study to effects observed among young people, which have been the focus of other research summarized below. Concerns have been raised that even if e-cigarettes are effective for net switching away from smoking among adults, they may act as a net gateway to smoking among adolescents. On the surface, it is not clear why the effect of e-cigarettes on smoking would completely reverse beginning at age 18 or 21. Nevertheless, analyses of longitudinal cohorts have concluded that adolescents who use e-cigarettes are more likely to report subsequent smoking. Crucially, this association is not necessarily causal and is reduced in analyses that adjust for more shared risk factors associated with use of both products among adolescents [4, 62]. Evidence of a gateway effect among young people is perhaps better explained by common liability or existing propensities (including social, environmental, and to some extent genetic [63]) to use tobacco products among other risky behaviours [64]. Criticisms of studies purporting to show a gateway effect from e-cigarette use to smoking among adolescents include inadequate adjustment for potential confounders [5] and lack of negative controls [65]. Recent meta-analyses have also criticized the gateway hypothesis, e.g. an upcoming Cochrane Tobacco Addiction Group review of e-cigarettes and subsequent smoking in young people concludes that there is only “very low” certainty of evidence for direct associations between e-cigarette use and initiation and progression of smoking [66]; and another recent meta-analysis identified “[n]umerous methodological flaws in the body of [gateway] literature [which] limit the generalizability of findings to the question of an association between e-cigarette use and cigarette smoking initiation” [67]. Finally, population-level research similar to the present study has found, contrary to prediction from gateway effects, that smoking prevalence among adolescents underwent an accelerated decline as e-cigarette use increased [9–18] and has stayed at historic lows in the latest National Youth Tobacco Survey data [19, 68]. Thus, trends among adults and adolescents at the population level appear to be consistent in this regard [44].

Important limitations must be noted: this study is ecological using cross-sectional data; therefore, causality cannot be established on these findings alone. Cross-sectional data also have selection and response bias, which may limit the accuracy of prevalence point estimates. Due to limitations in the questionnaire design of NHIS, the temporal ordering of product use (i.e., e-cigarettes being used before or after cigarette smoking) cannot be ascertained. As noted, there were some changes to survey methodology over time (accounted for by sensitivity testing). Finally, some of the observed decline in smoking may be due to other factors besides the FSPTCA, Tips^®^ campaign, and e-cigarette use, though the latter three are hypothesized to have had the greatest impact on smoking prevalence in the last decade.

Strengths of the analysis include the use of well-validated and representative population data over many years, and extensive sensitivity testing to establish the robustness of the model and findings across a range of assumptions, including different specifications of the cut-off year for the beginning of the e-cigarette era. The finding that the results apply to multiple sub-populations that have demonstrated substantial uptake of e-cigarettes also lends further confidence to the conclusions.

## Conclusions

Nationally representative population-level data on tobacco product use by US adults continue to support the existence of an association between increasing prevalence of e-cigarette use and decreasing prevalence of cigarette smoking, i.e., possible substitution between cigarettes and e-cigarettes.

### Author note

This article updates a previous report [22] with the most recent available data and with additional sensitivity tests. Over objections from the authors and others [69], the publisher elected to retract that prior article, based on a concern voiced by an unnamed member of their editorial board over the cut-off year between the pre- and e-cigarette eras, despite the fact that the paper itself explored a range of possible cut-offs, and further sensitivity analyses were provided in response to the editorial board member’s concern [70, 71].﻿ We have provided further details in comments on PubPeer at https://www.pubpeer.com/publications/0C19CEA0C329F1C95FC0884C7A4AE1, and we have made all correspondence with the previous journal and publisher available on OSF at 10.17605/OSF.IO/FZTNK.

### Electronic supplementary material

Below is the link to the electronic supplementary material.

Supplementary Material 1

## Data Availability

The datasets supporting the conclusions of this article are freely available in the National Health Interview Survey (NHIS) repository, https://www.cdc.gov/nchs/nhis/.

## Abbreviations

CDC: US Centers for Disease Control and Prevention
FSPTCA: Family Smoking Prevention and Tobacco Control Act
NHIS: National Health Interview Survey
RMSE: Root Mean Square Error
