# Supplementary material for: Increased e-cigarette use prevalence is associated with decreased smoking prevalence among US adults
Source: Harm Reduct J. 2024 Jul 18;21:136. doi: 10.1186/s12954-024-01056-0 (PMC11256395; doi:10.1186/s12954-024-01056-0)
Supplement: Supplementary file 1 — Supplementary Material 1 [file 12954_2024_1056_MOESM1_ESM.docx]

**Additional File 1**

**NHIS Questionnaire E-Cigarette Definitions**

In 2014, e-cigarettes were defined as “electronic cigarettes, often called e-cigarettes. E-cigarettes look like regular cigarettes, but are battery-powered and produce vapor instead of smoke.”

From 2015–2018, e-cigarettes were defined as “vape-pens, hookah-pens, e-hookahs, or e-vaporizers. Some look like cigarettes, and others look like pens or small pipes. These are battery-powered, usually contain liquid nicotine, and produce vapor instead of smoke… E-cigarettes and similar products can be bought as one-time, disposable products, as re-usable kits with a cartridge, or with refillable chambers. These usually contain a liquid, often called an “e-liquid” or “e-juice.” Popular brands include NJOY, BLU, LOGIC, and VUSE.”

In 2019, e-cigarettes were defined as “Electronic cigarettes (e-cigarettes) and other electronic vaping products include electronic hookahs (e-hookahs), vape pens, e-cigars, and others. These products are battery-powered and usually contain nicotine and flavors such as fruit, mint, or candy… These questions concern electronic vaping products for nicotine use. The use of electronic vaping products for marijuana use is not included in these questions.”

From 2020–2022, e-cigarettes were defined as “Electronic cigarettes (e-cigarettes) and other electronic vaping products include JUULs, vape pens, e-cigars, and others. These products are battery-powered and usually contain nicotine and flavors such as fruit, mint, or candy… These questions concern electronic vaping products for nicotine use. Do not include marijuana use.”

**Considerations Related to the Impacts of the FSPTCA and Tips Campaign**

Rossheim et al. [1] estimated that the FSPTCA was associated with a 0.6% reduction in US adult smoking prevalence each quarter from June 2009, or an approximate 31.2% reduction in smoking prevalence from mid-2009 to mid-2022 (0.6% times 52 quarters and conservatively assuming that the impact of the FSPTCA did not decline at all over time). Since NHIS smoking prevalence among all adults was 20.6±0.4% in 2009 (Figure 1 of the main article), applying Rossheim et al.’s 31.2% reduction gives a predicted smoking prevalence of 14.2% in 2022 from FSPTCA effects.

Similarly, Murphy-Hoefer et al. [2] estimated that the Tips® campaign was associated with 1 million sustained cigarette smoking quits from 2012–2018, or an approximate 0.4 percentage point decline in smoking prevalence (the US adult population was ~258.3 million in the 2020 Census [3], and 1/258.3≈0.004). Subtracting Murphy-Hoefer et al.’s 0.4 percentage point decline from Rossheim et al.’s 14.2% prevalence gives an expected smoking prevalence among all adults in 2022 of 13.8%, combining the impacts of the FSPTCA and Tips® campaign.

The actual observed NHIS smoking prevalence among all adults in 2022 was 11.6±0.3% (Figure 1 of the main article). Since the expected smoking prevalence from FSPTCA and Tips® campaign effects is greater than the actual observed smoking prevalence by 2.2 percentage points (13.8%-11.6%=2.2%), even *extremely* optimistic estimates of the FSPTCA and Tips® campaign effects combined are unable to fully explain the observed smoking prevalence in NHIS.

Furthermore, the estimates from Rossheim et al. [1] likely *overestimate* the impact of the FSPTCA because their analysis failed to account for the possibility that *any* other factor besides the flavored cigarette ban was responsible for the declines in smoking they observed, making our analyses even more conservative (i.e., working *against* an association between e-cigarette use and smoking). Rossheim et al. [1] actually showed that the impact of the flavored cigarette ban was strongest immediately after the ban and diminished thereafter, whereas here we conservatively assumed that the impact of these interventions were constant (i.e., did not diminish) and sustained (i.e., did not end) in every year.

**References**

1. Rossheim ME, Livingston MD, Krall JR, Barnett TE, Thombs DL, McDonald KK, Gimm GW: **Cigarette Use Before and After the 2009 Flavored Cigarette Ban**. *J Adolesc Health* 2020, **67**(3):432-437.

2. Murphy-Hoefer R, Davis KC, King BA, Beistle D, Rodes R, Graffunder C: **Association Between the Tips From Former Smokers Campaign and Smoking Cessation Among Adults, United States, 2012-2018**. *Prev Chronic Dis* 2020, **17**:E97.

3. **The U.S. Adult and Under-Age-18 Populations: 2020 Census** [<https://www.census.gov/library/visualizations/interactive/adult-and-under-the-age-of-18-populations-2020-census.html>]
